# Apoplastic recognition of multiple candidate effectors from the wheat pathogen *Zymoseptoria tritici* in the nonhost plant *Nicotiana benthamiana*

**DOI:** 10.1111/nph.14215

**Published:** 2016-10-03

**Authors:** Graeme J. Kettles, Carlos Bayon, Gail Canning, Jason J. Rudd, Kostya Kanyuka

**Affiliations:** ^1^ Department of Plant Biology & Crop ScienceRothamsted ResearchHarpendenHertfordshireAL5 2JQUK

**Keywords:** effector, *Mycosphaerella graminicola*, *Nicotiana benthamiana* (tobacco), nonhost resistance (NHR), receptor‐like kinase (RLK), *Septoria tritici*, virus‐induced gene silencing (VIGS), *Zymoseptoria tritici*

## Abstract

- The fungus *Zymoseptoria tritici* is a strictly apoplastic, host‐specific pathogen of wheat leaves and causal agent of septoria tritici blotch (STB) disease. All other plants are considered nonhosts, but the mechanism of nonhost resistance (NHR) to *Z. tritici* has not been addressed previously. We sought to develop *Nicotiana benthamiana* as a system to study NHR against *Z. tritici*.
- Fluorescence microscopy and quantitative reverse transcription polymerase chain reactions were used to establish the interaction between *Z. tritici* and *N. benthamiana*. Agrobacterium‐mediated transient expression was used to screen putative *Z. tritici* effector genes for recognition in *N. benthamiana*, and virus‐induced gene silencing (VIGS) was employed to determine the role of two receptor‐like kinases (RLKs), NbBAK1 and NbSOBIR1, in *Z. tritici* effector recognition.
- Numerous *Z. tritici* putative effectors (14 of 63 tested) induced cell death or chlorosis in *N. benthamiana*. For most, phenotypes were light‐dependent and required effector secretion to the leaf apoplastic space. Moreover, effector‐induced host cell death was dependent on NbBAK1 and NbSOBIR1.
- Our results indicate widespread recognition of apoplastic effectors from a wheat‐infecting fungal pathogen in a taxonomically distant nonhost plant species presumably by cell surface immune receptors. This suggests that apoplastic recognition of multiple nonadapted pathogen effectors may contribute to NHR.

## Abstract 2

See also the Commentary on this article by Friesen, **213:** 7–9.

## Introduction

Plants are able to resist infection or colonization by the majority of would‐be pathogens and pests they encounter, including bacteria, fungi, oomycetes, viruses and invertebrate pests. The fact that disease is relatively rare, suggests that plants have highly effective and robust means of repelling the vast majority of microbes they encounter. This most durable form of plant defence is termed nonhost resistance (NHR) and describes the resistance of a potential host to all isolates/races of a potential pathogen. However, despite its importance, our understanding of the mechanisms underpinning NHR in most cases remains poor, partly due to a lack of tractable model systems.

For plant‐associated microbes to become pathogens, they must evolve mechanisms to overcome or suppress the multi‐layered plant immune system. A multi‐phase framework for modelling plant immunity (primarily against biotrophic pathogens) has been described previously (Jones & Dangl, 2006). Plants perceive conserved molecular signatures termed pathogen‐associated molecular patterns (PAMPs) which leads to PAMP‐triggered immunity (PTI). Successful pathogens overcome PTI largely through the action of proteinaceous effectors or other metabolites which dampen the immune response and allow colonization. However, direct recognition of some pathogen effectors or their actions by plant disease resistance (R) proteins represents a second level of defence termed effector‐triggered immunity (ETI). This is often characterized by a localized host cell death (known as the hypersensitive response, HR) that is thought to spatially restrict or encase pathogens. For necrotrophic pathogens such as *Parastagonospora nodorum* and *Pyrenophora tritici‐repentis*, there is the additional activity of necrotrophic effectors (also known as host‐selective toxins) which operate via a model described as inverse gene‐for‐gene (Oliver & Solomon, 2010). For these interactions, immune recognition of an effector leads to host susceptibility rather than resistance.

A unifying hypothesis linking PTI/ETI to NHR has recently been suggested (Schulze‐Lefert & Panstruga, 2011). This hypothesis proposes that when little evolutionary distance separates host and nonhost, NHR is primarily dependent on the ETI branch of plant immunity. By contrast, larger evolutionary distances between host and nonhost rely more heavily on PTI, as nonadapted effectors are unable to successfully suppress PTI in the nonhost as they are in the host.

The current best‐studied systems for plant NHR have utilized obligate biotrophic fungi which penetrate plant cells physically at some point during infection and form haustorial‐like feeding structures within living cells of susceptible plants. Several studies have described *penetration* (*pen*) mutants of the model plant *Arabidopsis thaliana* which can be colonized by the normally nonpathogenic barley powdery mildew fungus, *Blumeria graminis* f. sp. *hordei* (Collins *et al*., 2003; Lipka *et al*., 2005; Stein *et al*., 2006). These mutants are impaired in ability to resist the direct penetration of the fungus, and callose deposition, glucosinolate activation, host membrane fusion and targeted focal secretion at attempted invasion sites all contribute towards NHR (Collins *et al*., 2003; Lipka *et al*., 2005; Stein *et al*., 2006; Bednarek *et al*., 2009; Clay *et al*., 2009). Barley (*Hordeum vulgare*) is itself a nonhost to the majority of nonadapted rusts, but is of intermediate status (at the seedling stage) to a small number of *Puccinia* rust species (Atienza *et al*., 2004). Accumulation of susceptibility alleles in single barley genotypes produced the SusPtrit and SusPmur lines, which are as susceptible to *Puccinia triticina* and *P. hordei‐murini* as the natural hosts, wheat (*Triticum aestivum*) and wall barley (*Hordeum murinum*), respectively (Atienza *et al*., 2004). Subsequent genetic mapping of quantitative trait loci (QTLs) associated with NHR revealed it to involve numerous genes from both host and pathogen, and to be highly variable depending on the pathogen (Jafary *et al*., 2006, 2008).

Arabidopsis also has been used as a model for NHR against several rust species (Bettgenhaeuser *et al*., 2014). Whilst rusts frequently germinate on Arabidopsis, they often fail to successfully penetrate stomata and colonize the substomatal cavity (Shafiei *et al*., 2007; Cheng *et al*., 2013). NHR to *P. triticina* was independent of classical phytohormone defence pathways, and QTLs associated with resistance contained genes encoding nucleotide‐binding site and leucine‐rich repeat (NB‐LRR or NLR) and receptor‐like kinase (RLK) proteins, two classes of genes implicated in plant disease resistance (Shafiei *et al*., 2007). Arabidopsis *pen* mutants did not display enhanced susceptibility to *P. triticina* (Shafiei *et al*., 2007), but did permit increased mesophyll colonization by the Asian soybean rust *Phakopsora pachyrhizi* (Loehrer *et al*., 2008), indicating that components of NHR effective against mildews may not be functional against some rust species.

*Zymoseptoria tritici* (previously known as *Mycosphaerella graminicola* or *Septoria tritici*) is a wheat leaf‐infecting Dothidiomycete fungus that displays near‐absolute host and tissue specificity. After germination of spores on the wheat leaf surface, it invades through stomata and remains exclusively in the leaf apoplastic space throughout its life cycle (Kema *et al*., 1996). Infection is typically symptomless for a period of 7–10 d before the appearance of disease symptoms, indicating that the fungus has transitioned to the necrotrophic phase of the life cycle. The fungus feeds on the nutrients released by dying plant cells, facilitating reproduction which is characterized by the development of pycnidia in sub‐stomatal cavities and the moisture‐triggered release of pycnidiospores that initiate further rounds of infection. This strictly extracellular (non‐plant cell penetrating) mode of colonization distinguishes *Z. tritici* from the current model systems used to study plant NHR against fungi, and opens up the question: How might NHR actually be achieved for pathogens that do not penetrate plant cells physically?

The molecular interactions between *Z. tritici* and wheat have recently received increased attention (Kettles & Kanyuka, 2016). As with all would‐be plant pathogens, *Z. tritici* is predicted to produce a diverse array of small secreted proteins throughout the interaction with host plants (Morais do Amaral *et al*., 2012; Mirzadi Gohari *et al*., 2015; Rudd *et al*., 2015). Several proteins have been described that contribute to *Z. tritici* pathogenesis on wheat. *Z. tritici* produces three LysM domain‐containing proteins (Mg1LysM, Mg3LysM, MgxLysM) two of which (Mg1LysM, Mg3LysM) have chitin‐binding properties (Marshall *et al*., 2011). Mg3LysM is a virulence factor and a functional orthologue of Ecp6 from the tomato mould *Cladosporium fulvum* (de Jonge *et al*., 2010; Mentlak *et al*., 2012) and is able to suppress chitin‐induced defence responses in wheat (Marshall *et al*., 2011). The Necrosis and Ethylene‐inducing Peptide 1 (NEP1)‐like protein (NLP) family are found in numerous plant pathogens. *Z. tritici* has a single NLP gene, *MgNLP*, which induces defence responses and cell death in dicotyledonous plants (Motteram *et al*., 2009) although not in wheat. *Z. tritici* mutants lacking functional *MgNLP* display no loss of virulence on wheat (Motteram *et al*., 2009), therefore its role in wheat infection is unknown. Recently, there has been the first description of necrotrophic effectors from *Z. tritici* (M'Barek *et al*., 2015). Necrosis‐inducing Proteins 1 & 2 (ZtNIP1, ZtNIP2) induce cell death and chlorosis, respectively, on some wheat cultivars, although the mechanism and contribution to virulence is not yet known.

To date, studies aimed at dissecting the molecular nature of *Z. tritici* virulence have exclusively utilized the fungus’ sole natural host, wheat. However, why *Z. tritici* fails to cause disease on any other nonhost plants has been unexplored to date. In the present work, we have developed the model tobacco species *Nicotiana benthamiana* (*N. benthamiana*) as a tool for the study of candidate *Z. tritici* effector proteins. By implementing a high‐level transient Agrobacterium‐mediated protein expression system, we found that recognition of *Z. tritici* candidate effectors is surprisingly common in this nonhost species. We report characterization of a subset of these effectors and their recognition in *N. benthamiana*. We also present evidence that perception of some *Z. tritici* effectors in this nonhost plant occurs at the plasma membrane‐apoplast interface, and is dependent on the previously described plant defence associated RLKs, NbBAK1 and NbSOBIR1.

## Materials and Methods

### Plant growth conditions

*Nicotiana benthamiana* seeds were germinated in Levington F2 + S compost (Everris Ltd, Ipswich, UK) in a 7 cm × 7 cm ×9 cm plastic pot kept in a humid chamber in an air‐conditioned glasshouse at 22°C : 18°C (day : night) with a 16 h photoperiod, with supplemental lighting where required. Seedlings were transplanted into individual 5 cm × 5 cm × 8 cm plastic pots when 2 wk old and returned to the same growth conditions. Plants were used for all experiments when 5–6 wk old.

### Fungal growth conditions and inoculations

Wild‐type *Zymoseptoria tritici* isolate IPO323 (Goodwin *et al*., 2011) and the transgenic green fluorescent protein (GFP)‐expressing B3 strain (Rohel *et al*., 2001) were grown on YPD agar plates for 4–5 d at 16°C. Conidiospores were harvested from plates and resuspended in distilled H_2_O + 0.01% Tween‐20. Spore concentrations were assessed by haemocytometer and standardized at 1 × 10^7^ spores ml^−1^. Spore solutions were either infiltrated into leaves using a 1 ml needleless syringe, or spray inoculated onto the leaf surface using an atomiser spray bottle.

### Confocal microscopy

The *Z. tritici* B3 isolate was spray‐inoculated onto the leaf surface of both *Triticum aestivum* and *N. benthamiana*. At 8 d post infiltration (dpi), 0.5 cm × 2 cm segments of infected leaves were cut using a razor blade and mounted on standard glass microscope slides under coverslips. Inverted leaf segments were viewed under a Zeiss 780LSM laser confocal microscope with 488 and 633 nm lasers and a 493–598 nm emission filter to capture both chlorophyll auto‐fluorescence and GFP excitation. Exposure settings were kept the same between all leaf samples.

### Cloning of candidate *Z. tritici* effectors

Sixty‐three full‐length (+ SP) candidate effectors (Supporting Information Tables S1, S2) were PCR‐amplified from cDNA derived from a *Z. tritici* isolate IPO323‐infected susceptible wheat leaf (cv Riband). AttB‐flanked PCR products were cloned into the Gateway‐compatible entry vector pDONR207 using BP clonase II enzyme mix (Thermo Fisher Scientific, Paisley, UK) following the manufacturer's instructions. Constructs were subsequently verified by Sanger sequencing (Eurofins Genomics, Wolverhampton, UK). Sequence‐verified candidate effector genes were recombined into either pEAQ‐HT‐DEST3 or pEARLEYGATE101 binary vectors (Earley *et al*., 2006; Sainsbury *et al*., 2009) using the LR clonase II enzyme mix (Thermo Fisher Scientific). All cloning steps were done using either DH5α or One Shot TOP10 (Thermo Fisher Scientific) electrocompetent *E. coli* cells following standard procedures. Effector genes that triggered cell death in *N. benthamiana* were subsequently recloned into the same vectors without the native secretion signal (−SP) as predicted by SignalP 4.0 (Petersen *et al*., 2011).

### Agrobacterium‐mediated expression of *Z. tritici* effectors

Agrobacterium strain GV3101 was transformed by electroporation with pEAQ‐HT‐DEST3 or pEARLEYGATE101 constructs containing candidate effector genes and cultured in LB supplemented with gentamicin (25 μg ml^−1^) and kanamycin (50 μg ml^−1^) at 28°C for 40 h. Cells were harvested by centrifugation and washed twice in infiltration buffer (10 mM MgCl_2_, 10 mM MES, pH 5.6) before final resuspension at OD_600_ = 1.2. Acetosyringone was added to a final concentration of 150 μM and cultures were incubated in the dark for 3 h before infiltration. Fully expanded leaves on 5–6‐wk‐old *N. benthamiana* plants were pressure‐infiltrated with a 1‐ml needleless syringe. For experiments to test light requirement for cell death, one Agrobacterium infiltrated leaf on each plant was kept under normal glasshouse light conditions whilst the other was wrapped in aluminium foil immediately following infiltration and remained shielded from light for the duration of the experiment. *Agrobacterium tumefaciens* strains containing pEARLEYGATE101 constructs were co‐infiltrated with a second *A. tumefaciens* strain expressing the p19 silencing suppressor protein of *Tomato bushy stunt virus* to enhance transient expression level.

### Virus‐induced gene silencing (VIGS) in *N. benthamiana*

All silencing constructs described use the *Tobacco rattle virus* (TRV)‐based vector PTV00 (Ratcliff *et al*., 2001). The *A. tumefaciens* strains harbouring PTV00:*GFP* and PTV00:*NbBAK1* have been described previously (Bos *et al*., 2006; Heese *et al*., 2007). For PTV00:*NbSOBIR1*, the silencing fragment described by Liebrand *et al*. (2013) was PCR amplified using primers SpeI NbSobir1 VIGS F and HindIII NbSobir1 VIGS R and digested using *Spe*I/*Hin*dIII. The fragment was inserted into *Spe*I/*Hin*dIII‐digested PTV00 using T4 DNA Ligase and verified by Sanger sequencing.

For all VIGS experiments, *A. tumefaciens* strains were grown for 40 h at 28°C in LB supplemented with appropriate antibiotics. All *A. tumefaciens* strains carrying PTV00 (TRV RNA2) derived constructs were prepared to OD_600_ = 1 and mixed at a 1 : 1 ratio with *A. tumefaciens* GV3101 strain carrying pBINTRA6 (TRV RNA1) also at OD_600_ = 1. Single leaves of 2–3‐wk‐old *N. benthamiana* seedlings were infiltrated using a 1‐ml needleless syringe. Upper uninoculated leaves of VIGS‐treated plants were infiltrated with *A. tumefaciens* carrying effector constructs at 2–3 wk after the initiation of virus infection. Effector‐induced cell death phenotypes were assessed visually at 7 dpi. Leaves were scored as positive for cell death if macroscopic symptoms were present at any point in the infiltrated zone. Leaves were scored as negative only if the entire infiltrated zone was free from symptoms. No distinction was made in severity of symptoms. For statistical evaluation, regression analysis was performed to assign variance attributable to VIGS treatment, effector treatment and experimental replicate within a generalized linear model (GLM) using Genstat (18^th^ edition, VSN International Ltd). The experimental replicate factor was standardized by averaging, thus allowing variance to be attributable to VIGS treatment and effector treatment. Tables showing least significant differences of predictions at three levels of significance (5%, 1%, 0.1%) are provided in (Table S3).

### Quantitative reverse transcription polymerase chain reaction (qRT‐PCR)

*Nicotiana benthamiana* leaf samples were ground in liquid nitrogen using a mortar and pestle and RNA extracted using Trizol (Invitrogen) following the manufacturer's instructions. 1–2 μg of RNA was DNase treated with the RQ1 DNase Kit (Promega) and cDNA was synthesized using SuperScript III (Thermo Fisher Scientific) reverse transcriptase. For *Z. tritici* effector expression analysis, undiluted cDNA was used. For analysis of *N. benthamiana* defence genes, cDNA was diluted 1 : 10 with dH_2_O. 1 μl of cDNA (neat or diluted) was used in each reaction with SYBR Green Jumpstart Taq ReadyMix (Sigma). Triplicate reactions were prepared for each sample/primer pair combination and all reactions were performed using the following thermocycle (2 min at 95°C followed by 45 cycles of 15 s at 95°C, 30 s at 60°C, and 35 s at 72°C). Relative expression values for either *N. benthamiana* defence genes or *Z. tritici* effector genes were calculated using the formula 2^−ΔCt^ relative to the *L23* (*N. benthamiana*) or β*‐tubulin* (*Z. tritici*) reference genes, respectively. For *N. benthamiana* defence gene induction, the mock treatment for each plant defence gene examined was rescaled to 1 for the purposes of presentation. For Z. *tritici* (Zt) effector gene expression, the inoculum sample for each gene examined was rescaled to 1 for presentation. Each experiment contained three biological replicates of each treatment, and each experiment was conducted twice with the same result. Primer sequences are provided in (Table S4).

**Figure 1:**
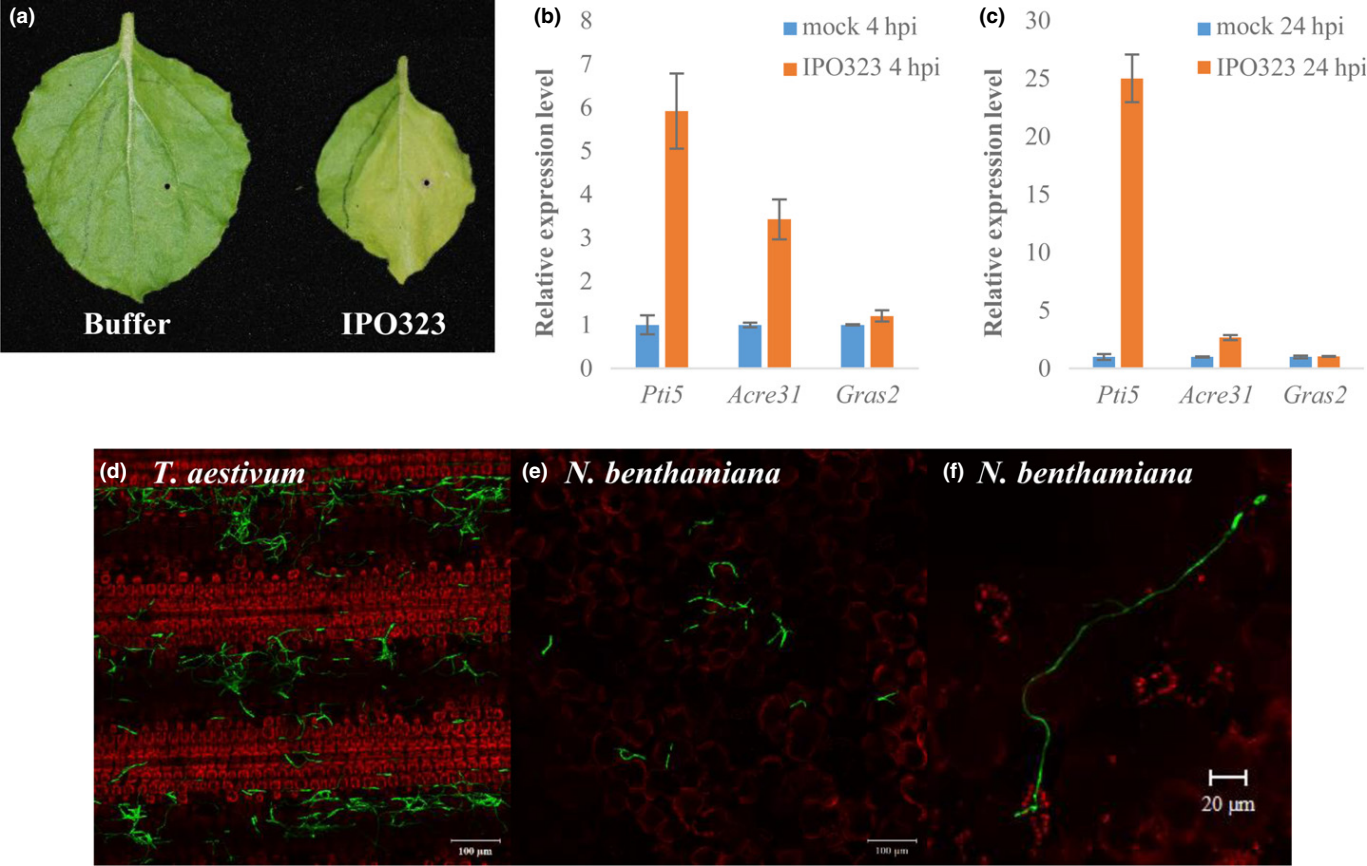
Nonhost interaction between *Nicotiana benthamiana* and *Zymoseptoria tritici*. (a) Symptom development following buffer‐only or *Z. tritici* isolate IPO323 spore infiltration (1 × 10^7^ spores ml^−1^) at 14 d post infiltration (dpi). (b, c) quantitative reverse transcription polymerase chain reaction (qRT‐PCR) analysis of PAMP‐triggered immunity (PTI)‐related genes at 4 h post infiltration (hpi) (b) and 24 hpi (c) following mock or fungal spore infiltration treatments. Bars represent means ± SE. Mock treatment rescaled to 1 for each defence gene assessed for presentation. (d–f) Typical growth of *Z. tritici* green fluorescent protein (GFP)‐expressing B3 strain following surface inoculation onto wheat (cv Riband) (d) and *N. benthamiana* (e) at 8 dpi. (f) Interaction between *Z. tritici* and *N. benthamiana* stomata was occasionally observed.

## Results

### *Zymoseptoria tritici* triggers plant defence responses during attempted infection of *N. benthamiana* leaves

We conducted several experiments to assess the interaction of both the reference isolate of *Z. tritici* IPO323 (Goodwin *et al*., 2011) and a strong constitutive GFP‐expressing strain (B3) (Rohel *et al*., 2001), with *N. benthamiana* leaves (Fig. 1). Infiltration of conidiospores of isolate IPO323 induced leaf chlorosis and inhibited leaf blade expansion and growth compared to control leaves of the same age treated only with the infiltration buffer (Fig. 1a). Chlorosis was first visible at 4–5 dpi, whilst inhibition of leaf growth took several further days to develop. However, neither macroscopic host cell death nor fungal pycnidia were visible in conidiospore‐infiltrated leaves when the experiment was terminated at 14 dpi, confirming that *Z. tritici* is unable to reproduce on *N. benthamiana*.

We subsequently used qRT‐PCR to assess the activation of plant defence genes previously implicated in PTI following conidiospore infiltration (Fig. 1b,c). The genes *Pti5*,* Acre31* and *Gras2* have been demonstrated previously to be responsive to both a nonadapted bacterium and a semi‐virulent bacterial pathogen in *N. benthamiana* (Nguyen *et al*., 2010). Expression of *Pti5* and *Acre31* was induced at both 4 h post infiltration (hpi) (Fig. 1b) and 24 hpi (Fig. 1c) with fungal conidiospores relative to the buffer‐only control. For *Pti5*, expression was higher at 24 hpi compared to 4 hpi, whereas *Acre31* expression was maintained at similar levels between the two time points. By contrast, *Gras2* was not induced at either time point (Fig. 1b,c), indicating that upregulation of this gene may be a specific feature of the PTI response to bacterial PAMPs (Nguyen *et al*., 2010).

In order to assess the behaviour of *Z. tritici* on the leaf surface, we spray‐inoculated leaves of both *N. benthamiana* and susceptible *T. aestivum* (cv Riband) with a conidiospore suspension of the GFP‐expressing B3 strain of *Z. tritici* (Rohel *et al*., 2001) (Fig. 1d–f). When leaves were assessed at 8 dpi, conidial germination was frequently observed on susceptible wheat leaves (Fig. 1d). By contrast, although GFP‐expressing spores were still visible at this time point on *N. benthamiana*, suggesting viable cells, hyphal growth was considerably less than on wheat (Fig. 1e). Nonetheless, the observation of some hyphal growth on *N. benthamiana* leaves indicates that the fungus attempted to colonize this nonhost species and is not immediately killed by pre‐formed chemical or physical defences. Interestingly, a minority of conidiospores formed considerably extended hyphae on *N. benthamiana* by 8 dpi, and interactions between hyphae and stomata were occasionally observed (Fig. 1f). However, our analysis to date has so far failed to provide firm evidence to confirm successful penetration of the sub‐stomatal cavity by *Z. tritici*. More detailed cytological analysis is required in future to ascertain the frequency of this occurrence. Overall, these data indicate that *N. benthamiana* can recognize and respond quickly to *Z. tritici*, that the fungus is viable on the leaf surface, but that overall hyphal growth is reduced and no pathogen reproduction is observed.

### Numerous *Z. tritici* effectors trigger cell death in the *N. benthamiana* leaf apoplast

**Figure 2:**
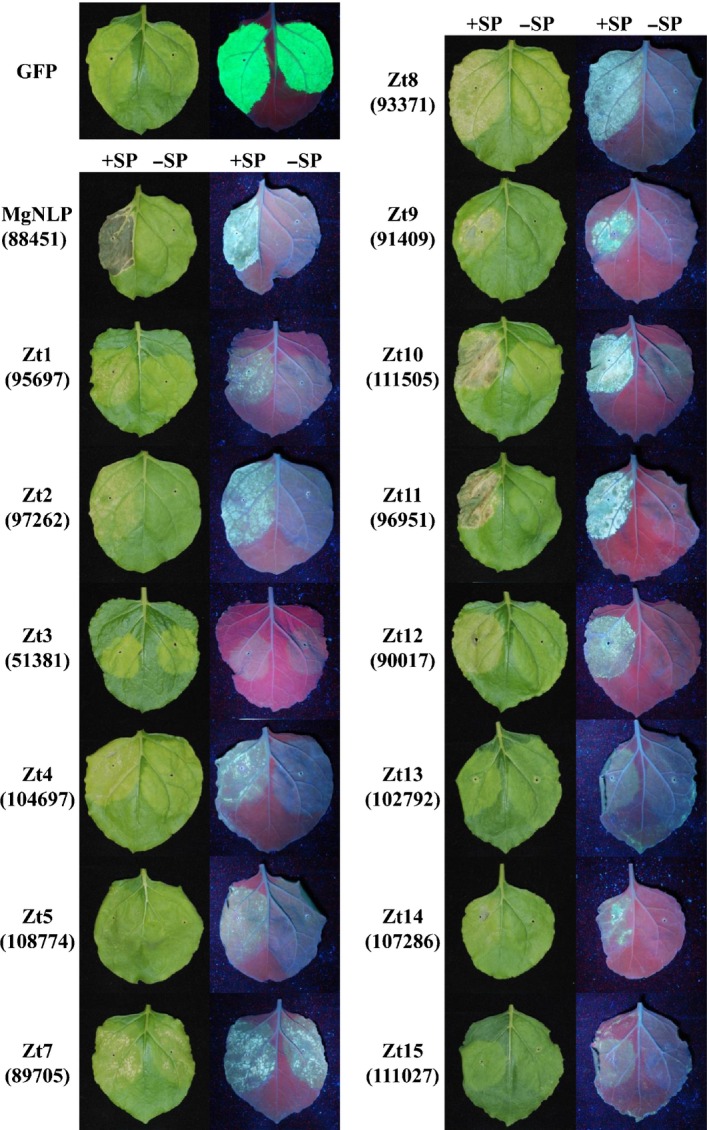
Agrobacterium‐mediated expression of *Zymoseptoria tritici* effectors in *Nicotiana benthamiana* induces localization‐dependent cell death or chlorosis. 14 (of 63 total) candidate effectors induced cell death or chlorosis phenotypes when expressed in *N. benthamiana* leaves. Expression of full‐length effectors containing secretion signal peptide (+ SP) compared to effectors lacking secretion signal peptide (−SP). Leaves photographed at 7 d post infiltration (dpi) and the same leaves shown under white and UV light for comparison.

During *Z. tritici* colonization of susceptible wheat, an initial phase of symptomless infection is followed by rapid transition to necrotrophy. Recent bioinformatic and transcriptomic analyses have identified large numbers of candidate effector proteins in the *Z. tritici* genome (Morais do Amaral *et al*., 2012; Yang *et al*., 2013; Mirzadi Gohari *et al*., 2015; Rudd *et al*., 2015; Palma‐Guerrero *et al*., 2016), many of which have characteristics similar to apoplastic effectors identified in other pathosystems. To test whether *Z. tritici* putative effectors are recognized in a nonhost plant, we established an Agrobacterium‐mediated transient effector expression system in *N. benthamiana*. This system utilizes the *Cowpea mosaic virus* (CPMV)‐derived pEAQ‐HT expression vector which facilitates high‐level and long‐lasting recombinant protein expression (Sainsbury *et al*., 2009). As a positive control for cell death induction in tobacco, we used the *Z. tritici* protein MgNLP described previously (Motteram *et al*., 2009). Consistent with previous results, MgNLP expressed using pEAQ‐HT also induced a strong cell death phenotype (Fig. 2) as indicated by macroscopic symptoms visible under white light and accumulation of cell death‐related auto‐fluorescent compounds visible under UV light. Moreover, this phenotype was observed only when *MgNLP* was cloned with its native secretion signal peptide (+ SP); expression of *MgNLP* lacking the secretion signal (−SP) did not induce cell death (Fig. 2). This is consistent with the apoplastic activity of this class of protein, and indicates that pEAQ‐HT is suitable for fungal effector expression in *N. benthamiana*. We therefore cloned 63 additional full‐length (+ SP) *Z. tritici* putative effectors into pEAQ‐HT for *in planta* expression. These were selected on the basis of being induced during early wheat leaf infection leading up to the transition to the necrotrophic growth phase (Rudd *et al*., 2015) (Tables S1, S2). Of these 63 effector constructs, 13 induced cell death of varying severity in *N. benthamiana* leaves (Zt1, Zt2, Zt4, Zt5, and Zt7–15) (Fig. 2). One of the effector constructs (Zt3) induced prominent chlorosis but not cell death (Fig. 2) as evidenced by an absence of accumulation of cell death‐related auto‐fluorescent phenylpropanoid compounds (Dixon & Paiva, 1995) when infiltrated leaves were viewed under UV light. No cell death was detected in GFP‐expressing control leaves (Fig. 2) or for the remaining 49 candidate effectors (not shown), indicating that the cell death phenotype is not due to the high and sustained level of transient protein expression. No cell death was observed from buffer‐only control infiltrations (not shown). The 14 *Z. tritici* candidate effectors that induced cell death or chlorosis also were cloned and expressed without signal peptides, to test the requirement for apoplastic localization for their induced phenotype (Fig. 2). Cell death‐inducing activity was either absent or considerably lower for all candidate effectors when expressed –SP compared to + SP, with the exception of Zt7, which displayed a similar phenotype irrespective of the presence or absence of the secretion signal (Fig. 2), and Zt3, which induced chlorosis independent of the presence or absence of the SP. These data indicate the presence of a surprisingly large number of proteins in the *Z. tritici* secretome that are recognized and result in cell death in the nonhost *N. benthamiana*. Additionally, the activity of most effectors identified (12 of 14) was dependent on direction to the apoplastic space in the infiltrated leaves.

In order to assess whether similar phenotypes could be observed when fungal effectors were expressed using a more common binary vector system, several genes (*GFP*,* MgNLP*,* Zt1*,* Zt9*) were re‐cloned into the nonviral pEARLEYGATE101 expression vector (Earley *et al*., 2006) for Agrobacterium‐mediated transient expression in *N. benthamiana*. This experiment revealed differences in the severity of phenotypes induced by transgene expression from the two vector systems (Fig. S1). GFP expression was stronger from pEAQ‐HT compared to pEARLEYGATE101 at both 3 and 7 dpi (Fig. S1a). The cell death phenotypes induced by MgNLP, Zt1 and Zt9 appeared noticeably weaker (MgNLP, Zt9) or were absent (Zt1) when these effectors were expressed from pEARLEYGATE101 compared to pEAQ‐HT (Fig. S1b). MgNLP, Zt1 and Zt9 proteins were detectable when expressed from pEARLEYGATE101 both with and without the SP, and the presence of secondary bands suggested these proteins may also be processed *in planta* (Fig. S2). Together, these data suggest that the pEAQ‐HT vector system may be particularly well‐suited to identifying less potent, potentially lower‐affinity interactions, or relatively unstable cell death inducers.

### Cell death induced by *Z. tritici* effectors is light dependent

**Figure 3:**
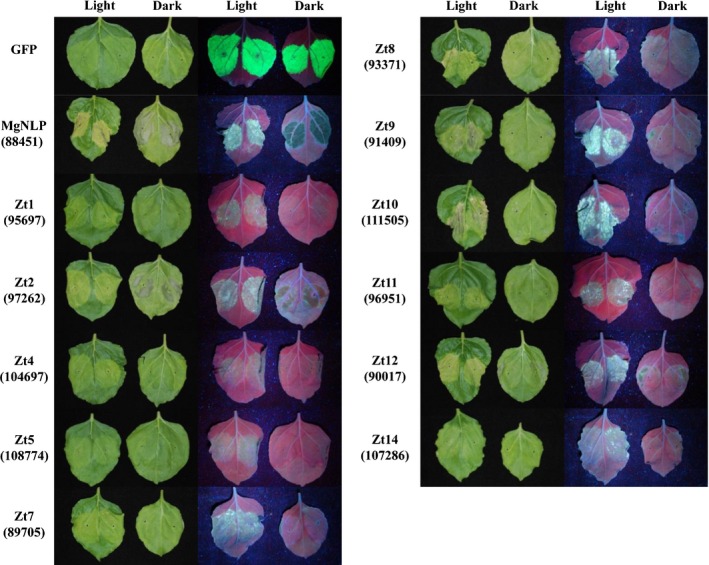
*Zymoseptoria tritici* effector‐induced cell death in *Nicotiana benthamiana* is light‐dependent. *Z. tritici* effectors (with signal peptide, + SP) were expressed in *N. benthamiana* using Agrobacterium‐mediated infiltration and leaves kept under a 16 h day–night cycle (Light) or in 24 h darkness (Dark) for 6 d. Two leaves from the same plant are shown for each effector treatment. The same leaves are shown under both white and UV light for comparison.

Using the transient overexpression system detailed above we identified 14 *Z. tritici* putative effector proteins with cell death/chlorosis‐inducing activity in *N. benthamiana* leaves. It has been demonstrated previously that light contributes to plant defence responses to pathogens and induction of HR or programmed cell death (PCD) (Asai *et al*., 2000; Brodersen *et al*., 2002; Zeier *et al*., 2004). Light‐dependency also is an important requirement for signalling triggered by necrotrophic fungal effectors (Manning & Ciuffetti, 2005; Liu *et al*., 2012). To test the requirement of light for effector‐induced cell death, full‐length (+ SP) effectors were transiently expressed as described previously, and leaves kept either under a normal day–night cycle (Light) or under complete darkness (Dark) for a period of 6 d (Fig. 3). Of 11 effectors that were tested in this experiment, nine were found to require light for full activity (Fig. 3), whilst only Zt2 and Zt12 induced cell death irrespective of light provision. Three effectors identified in the initial screen (Fig. 2) (Zt3, Zt13, Zt15) could not be assessed in this experiment, because dark treatment induced a leaf‐wide chlorosis that was too similar to the Zt3 phenotype, whilst the phenotypes induced by Zt13 and Zt15 were too weak to assess accurately. No difference in total GFP accumulation was observed when GFP‐expressing leaves were kept under both light regimes, indicating that light has minimal effect on the overall level of transgene expression for the duration of this experiment using the pEAQ‐HT vector system (Fig. 3). Surprisingly, MgNLP induced cell death in a light‐independent manner (Fig. 3), in contrast to the light‐dependency previously described for other members of the NLP class of effector (Qutob *et al*., 2006).

### Cell death‐inducing effectors are transcriptionally induced by *Z. tritici* when on the surface of *N. benthamiana* leaves

**Figure 4:**
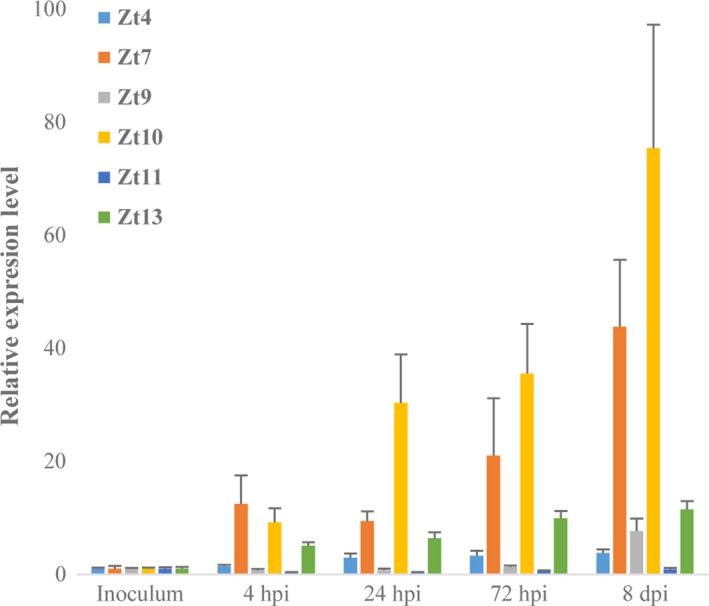
Expression profiling of *Zymoseptoria tritici* effector genes following inoculation onto *Nicotiana benthamiana* leaves. Quantitative reverse transcription polymerase chain reaction (qRT‐PCR) expression analysis of effector genes *Zt4, Zt7, Zt9, Zt10, Zt11* and *Zt13*. Leaves were spray‐inoculated with *Z. tritici* isolate IPO323 (1 × 10^7^ spores ml^−1^) and tissue samples harvested at the indicated time points. Expression of target fungal genes in inoculated leaf samples was then compared to the naive inoculum control. Bars represent mean + SE across three biological replicates from a single experiment. Expression level of each *Z. tritici* target gene in the inoculum sample rescaled to 1 for presentation. hpi, hours post infiltration; dpi, days post infiltration.

All cell death/chlorosis‐inducing *Z. tritici* effectors described in this work were previously found to be transcriptionally upregulated during *Z. tritici* growth on susceptible wheat leaves when compared to *in vitro* grown cultures (Rudd *et al*., 2015) (Fig. S3). The peak expression level of most also coincided with the fungal transition from symptomless to necrotrophic growth. We observed previously that when inoculated onto *N. benthamiana* leaves, a proportion of *Z. tritici* (isolate IPO323) conidiospores germinate and extend hyphae, although the extent of hyphal growth was considerably reduced compared to that on the surface of susceptible wheat leaves (Fig. 1d–f). To test whether candidate effectors are transcriptionally induced on *N. benthamiana*, leaves were spray‐inoculated with a *Z. tritici* IPO323 conidiospore inoculum and leaf samples harvested at several time points from 4 hpi to 8 dpi for qRT‐PCR analysis (Fig. 4). Of the six effector genes assessed, five showed considerable upregulation (*Zt4, Zt7, Zt9, Zt10, Zt13*) at multiple time points following inoculation onto *N. benthamiana*, compared to levels observed in the *in vitro* grown culture that served as inoculum (Fig. 4). The expression of *Zt4, Zt7, Zt10* and *Zt13* was higher across all four time points and was maximal at the final time point assessed (8 dpi). By contrast, *Zt9* was transcriptionally unchanged at three time points (4, 24 and 72 hpi) relative to the inoculum control, before increased expression was observed at 8 dpi (Fig. 4). The other effector gene tested, *Zt11*, was transcriptionally unresponsive at any time point relative to the inoculum control (Fig. 4), indicating that not all effector genes are similarly induced on nonhost leaves and that specific signals may be required to trigger particular genes. Nonetheless, these data indicate a broad similarity in the expression profile of cell death‐inducing effector genes on a nonhost (*N. benthamiana*) compared to the natural host (Rudd *et al*., 2015) (Fig. S3). These data also confirm our earlier observation of GFP‐expressing strains, which indicate that the fungus remains metabolically active and responsive for long periods whilst on the surface of tobacco leaves.

### Requirement for NbSERK3/BAK1 and NbSOBIR1 for *Z. tritici* effector‐induced cell death in *N. benthamiana*

**Figure 5:**
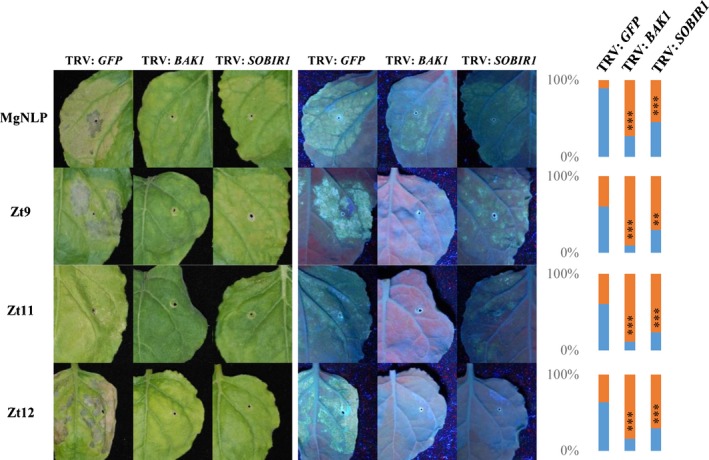
Cell death‐inducing activity of some *Zymoseptoria tritici* effectors is NbBAK1 and NbSOBIR1‐dependent. *Tobacco rattle virus* (TRV)‐based virus‐induced gene silencing (VIGS) vector PTV00 derivatives were used to initiate silencing of *NbBAK1* (TRV:*NbBAK1*) or *NbSOBIR1* (TRV:*NbSOBIR1*). TRV:*GFP* was used as a control virus treatment in these experiments. Agrobacterium‐mediated expression of effectors (+ SP) on systemically virus‐infected leaves conducted at 2–3 wk after initiation of VIGS. Agrobacterium‐infiltrated patches of leaves were scored for cell death and photos were taken at 7 d post infiltration (dpi). Panels on right show the proportion of leaves where cell death was present (blue) or absent (orange). Asterisks indicate differences determined by *t*‐probabilities within the GLM: **, *P *<* *0.01; ***, *P *<* *0.001.

The Brassinosteroid Insensitive 1 (BRI1)‐Associated Receptor Kinase 1 (BAK1) and the Suppressor of BIR1‐1 (SOBIR1) RLK proteins are important regulatory components required for functional immune signalling at the plasma membrane in several plant species (Liebrand *et al*., 2014). A principal function of these is to facilitate intracellular signalling following recognition of some extracellular PAMPs and apoplastic pathogen effectors by membrane‐bound RLKs and/or Receptor‐like Proteins (RLPs) (Heese *et al*., 2007; Liebrand *et al*., 2013, 2014; Albert *et al*., 2015). Our study indicated that most (12 of 13) cell death‐inducing effectors require localization to the leaf apoplastic space for activity in *N. benthamiana* (Fig. 2). One possibility is that effectors localized to the apoplastic space are recognized by unknown RLKs or RLPs that require NbBAK1 and/or NbSOBIR1 to initiate intracellular signalling that culminates in a cell death phenotype. To test whether these two RLKs are required for effector‐induced cell death in *N. benthamiana*, we used *Tobacco rattle virus* (TRV)‐mediated VIGS to knock‐down expression of *NbBAK1* or *NbSOBIR1* in *N. benthamiana* leaves. Young seedlings were inoculated with TRV:*NbBAK1* and TRV:*NbSOBIR1* VIGS constructs and candidate *Z. tritici* effectors subsequently were expressed using the pEAQ‐HT system in upper virus‐uninoculated leaves. The appearance of macroscopic symptoms of cell death were monitored in *NbBAK1*‐ or *NbSOBIR1*‐silenced leaves relative to leaves infected with a control virus (TRV:*GFP*) (Fig. 5). We tested three *Z. tritici* effectors (Zt9, Zt11, Zt12) that gave strong phenotypes in our original screen (Fig. 2) and found that cell death was almost completely absent in leaves of *NbBAK1*‐silenced plants relative to control plants (*P *<* *0.001, GLM) (Fig. 5). Cell death induced by the same three effectors was also less prevalent on *NbSOBIR1*‐silenced plants (*P *<* *0.001 for Zt11 and Zt12, *P *<* *0.01 for Zt9, GLM) (Fig. 5) although the difference in comparison to control plants was not as strong as observed for *NbBAK1*‐silenced plants. It has been demonstrated recently that immune signalling triggered by peptides derived from NLP‐class effector proteins requires complex formation involving both BAK1 and SOBIR1 (Albert *et al*., 2015). Consistent with this finding, cell death induced by *MgNLP* was significantly reduced in *NbBAK1*‐silenced plants compared to control plants (*P *<* *0.001, GLM). As observed for the three candidate effectors, there was a smaller but still highly significant reduction in the prevalence of cell death in leaves of *NbSOBIR1*‐silenced plants compared to control plants (*P *<* *0.001, GLM). Together, these data indicate an involvement of both NbBAK1 and NbSOBIR1 in cell death triggered by expression of three *Z. tritici* effector proteins in *N. benthamiana*.

## Discussion

*N. benthamiana* is well established as a model for studying effectors from dicot‐infecting pathogens, but has been used less frequently for cereal (monocot)‐infecting fungi. Petre and colleagues recently described expression of a small set of candidate effectors from the wheat yellow rust pathogen *Puccinia striiformis* f. sp. *tritici* in *N. benthamiana* (Petre *et al*., 2016). Nine candidate effectors were found to localize to distinct subcellular compartments, and protein–protein interactions studies identified a specific target for one of those effectors (Petre *et al*., 2016). However, in contrast with the data described here (Fig. 2), they did not report any incidence of cell death induced by any of the effectors studied. In another recent study, a number of effectors from the rice false smut pathogen *Ustilaginoidea virens* were reported to induce cell death in both *N. benthamiana* and rice (Fang *et al*., 2016). Similar to findings of our study, all of the cell death‐inducing *U. virens* effectors were shown to require secretion to induce phenotypes, both in *N. benthamiana* leaves and rice protoplasts. *U. virens* is a biotrophic pathogen of rice floral tissue, and its lifestyle is considerably different to that of *Z. tritici*. Numerous other *U. virens* effectors also have been shown to suppress pathogen‐induced HR in *N. benthamiana* (Zhang *et al*., 2014), a phenotype not demonstrated previously for any *Z. tritici* effectors.

Using the GFP‐expressing B3 strain of *Z. tritici*, we observed that fungal conidiospores are metabolically active up to 8 d post inoculation (dpi) onto the *N. benthamiana* leaf surface, and in some cases exhibit a substantial degree of hyphal growth. However, we were unable to detect successful penetration of the sub‐stomatal cavity. It is possible that chemical and physical cues on the *N. benthamiana* leaf surface might also contribute to the low rate of stomatal penetration, which may work in tandem with the contribution made via recognition of secreted effector proteins, to overall plant resistance. It is known that during the early symptomless phase of *Z. tritici* colonization of wheat, there is simultaneous upregulation of numerous secreted lipases and cutinases (Rudd *et al*., 2015). Whilst it is not known whether this also occurs on *N. benthamiana*, if so, there is the possibility that enzymatic cuticular degradation by *Z. tritici* may open a direct interface between secreted fungal proteins (including effectors) and host epidermal cell surface receptors. This interaction may partly explain the reduced rate of fungal growth on nonhost leaf surfaces, and ultimately, failure to penetrate stomata. Furthermore, recent demonstration of cell death‐inducing activity of the *Parastagonospora nodorum* effector SnTox1 on the wheat leaf surface (Liu *et al*., 2016) indicates that some effectors function before leaf penetration.

Of the 14 *Z. tritici* effectors that induce cell death or chlorosis in *N. benthamiana*, 13 have no putative function or conserved domains. The single exception is Zt3, which is a predicted glycoside hydrolase. For the remainder, there are varying degrees of similarity to putative proteins from other fungi or bacteria (Table S2). Seven effectors (Zt3, Zt4, Zt5, Zt9, Zt10, Zt12, Zt15) show similarity to proteins from the grass‐infecting fungus *Zymoseptoria brevis*, whilst two effectors (Zt11, Zt13) have no known orthologues in other species and appear unique to *Z. tritici*.

The cell death‐inducing effector Zt12 previously has been annotated as the *M. graminicola* Tandem Repeat Protein (MgTRP) MgTRP17 (Rudd *et al*., 2010). Mature Zt12 is 133 amino acids long and rich in both glycine (28.6%) and asparagine (21.1%). Of the 23 MgTRPs, Zt12 has the lowest Rapid Automatic Detection and Alignment tool (Heger & Holm, 2000) repeat score (Rudd *et al*., 2010), indicating that the repetitive unit is comparatively irregular. Therefore, Zt12 probably represents a glycine‐rich protein (GRP). GRPs previously have been implicated in plant immune processes (Mangeon *et al*., 2010). The Arabidopsis GRP AtGRP7 is targeted for ADP‐ribosylation by the *Pseudomonas syringae* effector HopU1, and Arabidopsis *grp7* mutants are more susceptible to infection by this bacterial pathogen than wild‐type plants (Fu *et al*., 2007). Furthermore, two other Arabidopsis GRPs (AtGRP3 and AtGRP3S) have been shown to function as endogenous extracellular ligands of the wall‐associated protein kinase 1 (WAK1) (Park *et al*., 2001) and AtGRP3 is able to induce expression of the defence gene *PR1*. In the present study (Fig. 5), we identified that Agrobacterium‐mediated expression of Zt12 triggers cell death in *N. benthamiana* in a manner that is dependent on the receptor‐associated RLKs NbBAK1 and NbSOBIR1. Therefore, it is likely that a currently unknown cell surface receptor recognizes Zt12, and initiates a signalling cascade dependent on NbBAK1 and NbSOBIR1, culminating in apoptosis‐like cell death in *N. benthamiana*. If this model is confirmed, it will be interesting for future investigations to test whether recognition of Zt12 is specific to *N. benthamiana* or is common to other nonhost plants.

Of the 14 *Z. tritici* effectors described here, only Zt3 induced chlorosis with no indication of obvious macroscopic cell death (Fig. 2). Zt3 is a putative glycoside hydrolase family 53 protein with predicted endo‐1,4‐β‐galactosidase activity against arabinogalactans, a component of plant cell walls. It has been speculated that arabinogalactan proteins (AGPs) may interact with WAKs based on colocalization in tobacco protoplasts (Gens *et al*., 2000). In addition, some AGPs contain the 6‐cysteine PAC domain, common to the tomato cysteine‐rich extracellular protein LAT52 and involved in perception by the pollen receptor kinase LePRK2 (Tang *et al*., 2002). Interestingly, the secreted protein XEG1 from the oomycete *Phytophthora sojae*, which belongs to the glycoside hydrolase family 12 with activity against xyloglucan and β‐glucan, recently was identified as an inducer of cell death in *N. benthamiana* (Ma *et al*., 2015).

Light requirement for progression of PCD has been documented for numerous plant–pathogen interactions, and also during reactions to individual effector proteins (Asai *et al*., 2000; Brodersen *et al*., 2002; Zeier *et al*., 2004; Manning & Ciuffetti, 2005; Liu *et al*., 2012). The majority of *Z. tritici* effectors described here also require light to induce cell death in *N. benthamiana*. However, two effectors (Zt2, Zt12) induced cell death in a light‐independent manner, suggesting that these two proteins may have distinct activities in this plant species. Disease progression during *Z. tritici* infection of wheat also has a light‐dependent component (Keon *et al*., 2007). Wheat leaves silenced for components of chlorophyll or carotenoid biosynthesis exhibit more rapid cell death during *Z. tritici* infection (Lee *et al*., 2014). However, these leaves are subsequently less able to support sporulation. Whether the effectors described here contribute to *Z. tritici* pathogenesis of wheat remains unknown. However, it seems reasonable to assume that given the light requirement for full disease progression, a subset of fungal effectors, either those identified here or others that remain to be found, may contribute to this phenomenon.

The tomato mould *C. fulvum* is a useful model for *Z. tritici* infection as both pathogens exist exclusively in the apoplastic space of host plants. Gene‐for‐gene interactions between *C. fulvum* and tomato are well‐described, in particular, recognition of the apoplastic effectors Avr2/Avr4/Avr9 by the corresponding RLPs Cf2/Cf4/Cf9. These interactions can also be reconstituted in *N. benthamiana*, facilitating identification of additional immune complex components. Recently, the membrane‐associated RLKs BAK1 and SOBIR1 were shown to be required for full immunity following Avr4 recognition by Cf4 (Liebrand *et al*., 2013; Postma *et al*., 2016). The data presented here (Fig. 5) suggest that a similar mechanism operates for *N. benthamiana* recognition of at least the three tested *Z. tritici* effectors (Zt9, Zt11, Zt12), where both NbBAK1 and NbSOBIR1 were required for effector‐triggered cell death. This requirement has not been demonstrated for effectors from either *P. striiformis* f. sp. *tritici* or *U. virens* (Fang *et al*., 2016; Petre *et al*., 2016). Therefore, we can speculate that such signalling may be hallmarks of effector recognition from apoplastic pathogens. However, in the case of *Z. tritici* effectors, the specific recognition determinants at the cell surface remain to be identified.

All of the 14 cell death‐inducing *Z. tritici* effectors displayed increased expression during infection of susceptible wheat leaves compared to *in vitro* cultured fungus (Rudd *et al*., 2015). Additionally, maximal expression of most candidate effectors occurred at 4 or 9 dpi, either before or during the fungal transition to necrotrophic growth. In the present study, we show that effector expression during attempted infection of *N. benthamiana* is also considerably higher than in the *in vitro* fungal culture (Fig. 4). Maximal expression of all but one of the candidate effector genes tested occurred at 8 dpi (Fig. 4), a comparable time point to an infection time course on wheat described previously (Rudd *et al*., 2015). These observations suggest that induction of particular effectors expression in planta following spore germination may be largely independent of the leaf substrate, or indeed whether there is successful stomatal penetration. Transcriptome data previously suggested that on the nutrient‐poor leaf surface, *Z. tritici* relies upon the mobilization of either or both extracellular and stored lipids for initial colonization (Rudd *et al*., 2015). It may be that induction of the effector repertoire during the same time points is a direct consequence of nutrient scarcity rather than detection of stimulatory host‐derived cues.

We initially identified cell death‐inducing activity of *Z. tritici* effectors using the plant virus‐derived pEAQ‐HT vector system (Sainsbury *et al*., 2009) (Fig. 2). We then utilized a second, conventional binary vector expression system (pEARLEYGATE101) (Earley *et al*., 2006) and found that effector‐induced phenotypes were weaker or occasionally absent from Agrobacterium‐infiltrated leaves (Fig. S1). In addition, detection of effectors expressed from pEARLEYGATE101 indicated that some may be unstable or extensively processed in planta (Fig. S2). The pEAQ‐HT vector system was originally developed for ultrahigh expression of heterologous proteins in plants and concentrations of accumulating GFP are higher from pEAQ‐HT than pEARLEYGATE101 (Fig. S1). It is therefore possible that putative effectors from *Z. tritici* are recognized in *N. benthamiana* with relatively low affinity or are unstable *in planta*, and require the sustained high‐level expression provided by the pEAQ‐HT system to progress to macroscopically visible cell death. *Z. tritici* displays absolute specificity for infecting wheat leaves, and occurs predominantly in temperate, maritime climates. By contrast, *N. benthamiana* is native to northern Australia and is unlikely to encounter *Z. tritici* in nature. Recognition determinants in *N. benthamiana* are highly unlikely to have evolved to specifically recognize *Z. tritici* effectors. However, weak or transient recognition can be amplified by use of the pEAQ‐HT system to reveal previously undetectable cell death phenotypes. This represents a novel use for this vector system which has not been used widely in studies of pathogen effectors. However, it is possible that its use may reveal many, potentially lower affinity, but still specific, recognition factors for multiple putative pathogen effectors.

A model uniting PTI/ETI, pathogen effectors and NHR was developed by Schulze‐Lefert & Panstruga (2011). The model proposes that with large evolutionary divergence between host and nonhost (such as *T. aestivum* and *N. benthamiana*) the contribution of ETI to resistance is minimal for two reasons: first, that nonadapted pathogen effectors are unable to ‘find’ natural targets as they are in the host; and second, that rapidly evolving host recognition determinants (NLRs) have lost capacity to recognize effectors from distant potential pathogens (Schulze‐Lefert & Panstruga, 2011). Our findings do not immediately fit within this model, as our data suggests that effector recognition in a distant nonhost might be relatively common in some nonhost interactions. However, it is possible that our novel use of the very high level pEAQ expression system may have permitted us to detect interactions that are normally of lower affinity, and may not be detected using other systems. Without further evidence, however, this remains speculative. Our data are not contradictory to the model, simply that an extension of the model might be required to accommodate interactions that may be specific to apoplastic pathogens with a host cell non‐penetrating mode of infection. In this case, effector recognition may largely occur at the cell surface, mediated by RLPs/RLKs, as opposed to intracellular recognition by NLRs, and that RLPs/RLKs may be subject to different evolutionary pressures than NLRs such that they retain ability to identify distant effectors.

The results presented here reveal recognition, and subsequent cell death, triggered by numerous effector proteins from *Z. tritici* in a nonhost plant. The function of these effectors, and indeed whether they are recognized in the fungus’ natural host remains unknown. However, it will be interesting to assess whether recognition of these candidate effector proteins (or perhaps others) is common in plants that are resistant to infection by *Z. tritici*. If so, the possibility emerges that recognition of multiple apoplastic effectors, perhaps through multiple lower affinity RLPs/RLKs, may contribute to nonhost resistance against this, and potentially other pathogens with this mode of colonization.

## Author contributions

J.R.R. and K.K. initially conceived the project; G.J.K., J.R.R. and K.K. designed the experiments; G.J.K., C.B. and G.C. conducted all experimental work; G.J.K. analysed experimental data; and G.J.K., J.R.R. and K.K. wrote the manuscript.

## Supporting information

Please note: Wiley Blackwell are not responsible for the content or functionality of any Supporting Information supplied by the authors. Any queries (other than missing material) should be directed to the *New Phytologist* Central Office.

**Fig. S1** Phenotype comparison of pEAQ‐HT and pEARLEYGATE101 vector systems.
**Fig. S2** Detection of effectors expressed from pEARLYEGATE101.
**Fig. S3** *Z. tritici* effector expression during infection timecourse of wheat.

**Table S1** List of 63 *Z. tritici* candidate effectors cloned and expressed in *N. benthamiana*

**Table S2** Standard protein Blast (Blastp) analysis of 14 *Z. tritici* effectors showing 5 best hits (when available) from NCBI Blast web service on 2^nd^ June 2016

**Table S3** Regression analysis of VIGS experimental data

**Table S4** Primer sequences used in this study
