# Supplementary material for: Apoplastic recognition of multiple candidate effectors from the wheat pathogen Zymoseptoria tritici in the nonhost plant Nicotiana benthamiana
Source: New Phytol. 2016 Oct 3;213(1):338–50. doi: 10.1111/nph.14215 (PMC5132004; doi:10.1111/nph.14215)
Supplement: Supplementary file 1 — Fig. S1 Phenotype comparison of pEAQ‐HT and pEARLEYGATE101 vector systems. Fig. S2 Detection of effectors expressed from pEARLYEGATE101. Fig. S3 Z. tritici effector expression during infection timecourse of wheat. [file NPH-213-338-s001.pdf]

New Phytologist

***New Phytologist* Supporting Information**

Article title: **Apoplastic recognition of multiple candidate effectors from the wheat pathogen *Zymoseptoria tritici* in the nonhost plant *Nicotiana benthamiana***

Authors: Graeme J. Kettles, Carlos Bayon, Gail Canning, Jason J. Rudd and Kostya Kanyuka

Article acceptance date: 19 August 2016

The following Supporting Information is available for this article:

Fig. S1 Phenotype comparison of pEAQ-HT and pEARLEYGATE101 vector systems.

Fig. S2 Detection of effectors expressed from pEARLYEGATE101.

Fig. S3 Zt effector expression during *Z. tritici* infection timecourse of wheat.

Table S1 List of 63 *Z. tritici* candidate effectors cloned and expressed in *N. benthamiana*.

Table S2 Standard protein BLAST (blastp) analysis of 14 *Z. tritici* effectors showing 5 best hits (if available) from NCBI BLAST web service on 2nd June 2016.

Table S3 Regression analysis of VIGS experimental data.

Table S4 Primer sequences used in this study.

Fig. S1 Phenotype comparison of pEAQ-HT and pEARLEYGATE vector systems. Genes were expressed from pEAQ-HT-DEST3 (pEAQ-HT) or pEARLEYGATE101 (pEG) and phenotypes assessed at 3 dpi or 7 dpi. pEG constructs were co-infiltrated with p19 to enhance expression.

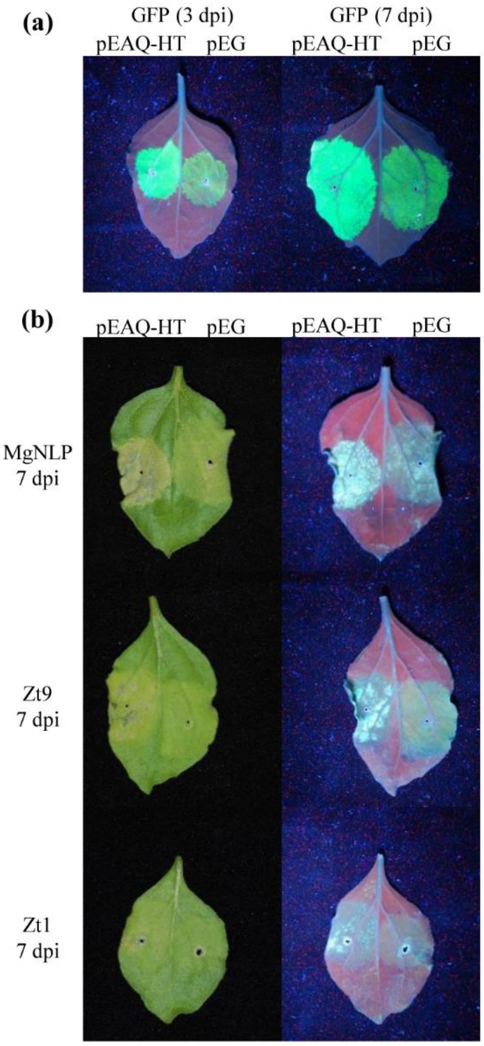

Fig. S2 Detection of *Z. tritici* effector proteins agroexpressed in *N. benthamiana*. GFP and three *Z. tritici* effectors (MgNLP, Zt1, Zt9) were agroexpressed in *N. benthamiana* leaves using the pEARLEYGATE101 vector. Effectors were expressed both with and without (-SP) secretion signal peptides. Leaves were harvested at 3 dpi and total and soluble protein extracts were prepared. Predicted sizes are; GFP-YFP-HA (59KDa), MgNLP-YFP-HA (56KDa), Zt9-YFP-HA (40KDa), Zt1-YFP-HA (43KDa).

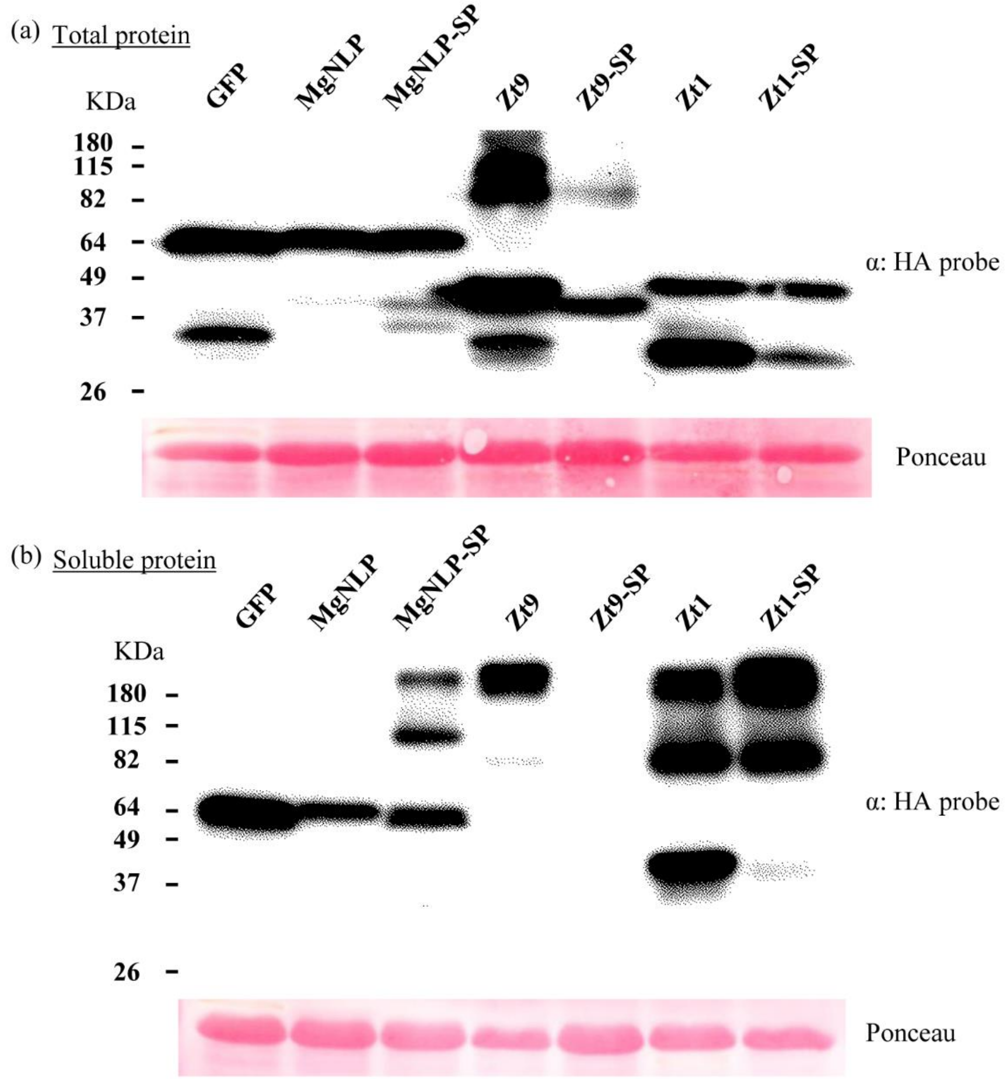

Fig. S3 Zt effector expression during *Z. tritici* infection timecourse of wheat.** RNAseq expression profiles of candidate effectors Zt4, Zt7, Zt9, Zt10, Zt11 and Zt13 during *Z. tritici* (IPO323) infection of susceptible wheat (cv. Riband) relative to in vitro growth in Czapek-Dox broth (CDB). All data taken from Rudd *et al.* (2015). For each gene, the published FPKM values were converted to relative expression values where the CDB sample is rescaled to 1 for presentation.

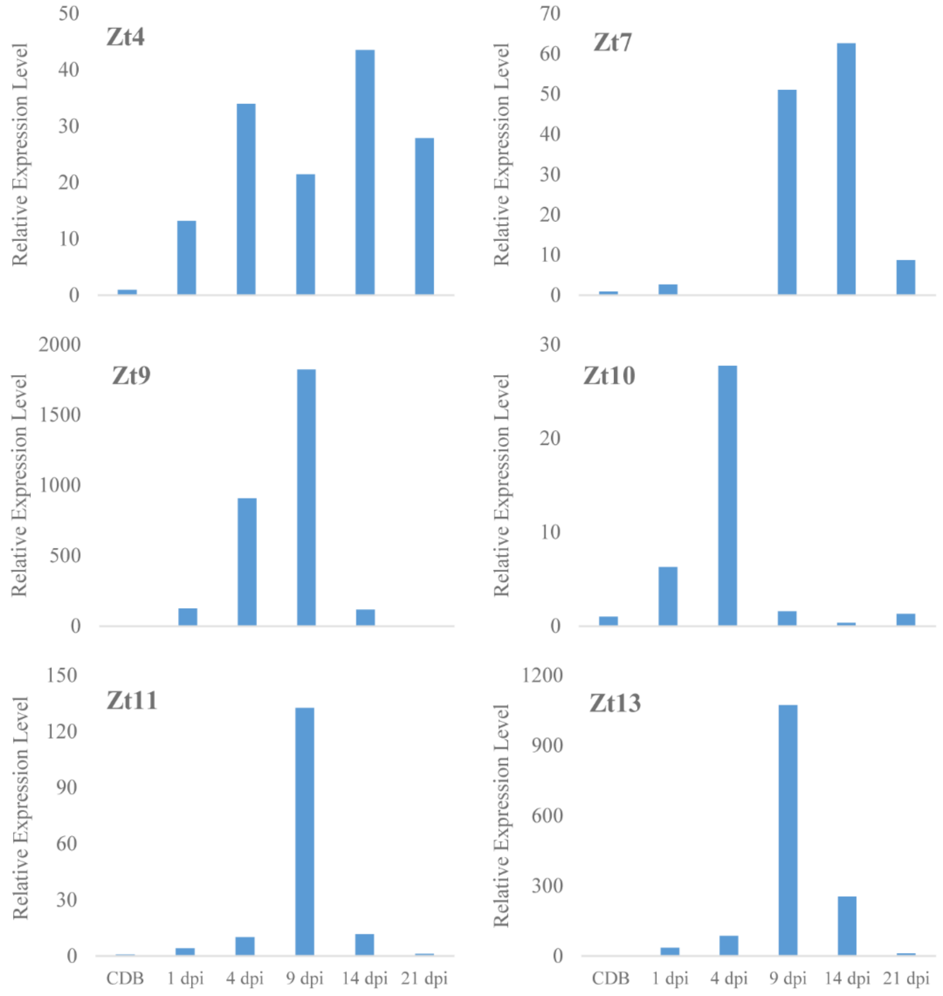

Table S1 List of 63 *Z. tritici* candidate effectors cloned and expressed in *N. benthamiana* (see separate file).

Table S2 Standard protein BLAST (blastp) analysis of 14 Zt effectors showing best 5 hits (if available) from NCBI BLAST web service on 2nd June 2016 (see separate file).

Table S3 Regression analysis of VIGS experimental data. Predictions from the regression model and least significant differences of predictions at three significance levels (5%, 1% and 0.1%) are shown (see separate file).

Table S4 Primer sequences used in this study (see separate file).

References

Rudd JJ, Kanyuka K, Hassani-Pak K, Derbyshire M, Andongabo A, Devonshire J, Lysenko A, Saqi M, Desai NM, Powers SJ *et al.* 2015. Transcriptome and metabolite profiling of the infection cycle of *Zymoseptoria tritici* on wheat reveals a biphasic interaction with plant immunity involving differential pathogen chromosomal contributions and a variation on the hemibiotrophic lifestyle definition. *Plant Physiology* **167**: 1158–1185.
