# Supplementary material for: Apoplastic recognition of multiple candidate effectors from the wheat pathogen Zymoseptoria tritici in the nonhost plant Nicotiana benthamiana
Source: New Phytol. 2016 Oct 3;213(1):338–50. doi: 10.1111/nph.14215 (PMC5132004; doi:10.1111/nph.14215)
Supplement: Supplementary file 4 — Table S3 Regression analysis of VIGS experimental data [file NPH-213-338-s004.rtf]

Predictions from regression model
 
These predictions are estimated mean proportions, formed on the scale of the response variable, corresponding to one binomial trial, adjusted with respect to some factors as specified below.
 
The predictions have been formed only for those combinations of factor levels that are present in the data.
 
The predictions have been standardized by averaging over the levels of some factors:
	Factor	Weighting policy	Status of weights
	experiment	Marginal weights	Adjusted to exclude combinations not present
 
The standard errors are appropriate for interpretation of the predictions as summaries of the data rather than as forecasts of new observations.
 
Response variate: death
 
	VIGS	GFP		BAK1	
		Prediction	s.e.	Prediction	s.e.
	Effector	 
	90017	0.6349	0.06807	0.1511	0.05221
	91409	0.6052	0.06832	0.0916	0.04340
	96951	0.6052	0.06832	0.1068	0.04487
	NLP	0.9048	0.04450	0.2951	0.09100
 
 
	VIGS	SOBIR1	
		Prediction	s.e.
	Effector	 
	90017	0.2764	0.06419
	91409	0.2948	0.06497
	96951	0.2314	0.06029
	NLP	0.4856	0.08562
 
 
 

Least significant differences of predictions (5% level)
 
		 	 	 	 	 
	Effector 90017 VIGS GFP	1	 *			
	Effector 90017 VIGS BAK1	2	 0.1745	 *		
	Effector 90017 VIGS SOBIR1	3	 0.1903	 0.1683	 *	
	Effector 91409 VIGS GFP	4	 0.1962	 0.1749	 0.1907	 *
	Effector 91409 VIGS BAK1	5	 0.1642	 0.1381	 0.1576	 0.1647
	Effector 91409 VIGS SOBIR1	6	 0.1914	 0.1695	 0.1858	 0.1918
	Effector 96951 VIGS GFP	7	 0.1962	 0.1749	 0.1907	 0.1965
	Effector 96951 VIGS BAK1	8	 0.1658	 0.1400	 0.1593	 0.1663
	Effector 96951 VIGS SOBIR1	9	 0.1850	 0.1622	 0.1791	 0.1853
	Effector NLP VIGS GFP	10	 0.1655	 0.1397	 0.1590	 0.1659
	Effector NLP VIGS BAK1	11	 0.2316	 0.2138	 0.2270	 0.2318
	Effector NLP VIGS SOBIR1	12	 0.2228	 0.2043	 0.2180	 0.2230
		 	1	2	3	4
 
		 	 	 	 	 
	Effector 91409 VIGS BAK1	5	 *			
	Effector 91409 VIGS SOBIR1	6	 0.1590	 *		
	Effector 96951 VIGS GFP	7	 0.1647	 0.1918	 *	
	Effector 96951 VIGS BAK1	8	 0.1270	 0.1606	 0.1663	 *
	Effector 96951 VIGS SOBIR1	9	 0.1511	 0.1803	 0.1853	 0.1529
	Effector NLP VIGS GFP	10	 0.1265	 0.1603	 0.1659	 0.1286
	Effector NLP VIGS BAK1	11	 0.2052	 0.2279	 0.2318	 0.2067
	Effector NLP VIGS SOBIR1	12	 0.1954	 0.2189	 0.2230	 0.1968
		 	5	6	7	8
 
		 	 	 	 	 
	Effector 96951 VIGS SOBIR1	9	 *			
	Effector NLP VIGS GFP	10	 0.1525	 *		
	Effector NLP VIGS BAK1	11	 0.2224	 0.2058	 *	
	Effector NLP VIGS SOBIR1	12	 0.2133	 0.1960	 0.2526	 *
		 	9	10	11	12
 
 

 
 

Least significant differences of predictions (1% level)
 
		 	 	 	 	 
	Effector 90017 VIGS GFP	1	 *			
	Effector 90017 VIGS BAK1	2	 0.2344	 *		
	Effector 90017 VIGS SOBIR1	3	 0.2556	 0.2261	 *	
	Effector 91409 VIGS GFP	4	 0.2635	 0.2350	 0.2562	 *
	Effector 91409 VIGS BAK1	5	 0.2206	 0.1855	 0.2117	 0.2212
	Effector 91409 VIGS SOBIR1	6	 0.2571	 0.2277	 0.2495	 0.2576
	Effector 96951 VIGS GFP	7	 0.2635	 0.2350	 0.2562	 0.2640
	Effector 96951 VIGS BAK1	8	 0.2228	 0.1881	 0.2140	 0.2234
	Effector 96951 VIGS SOBIR1	9	 0.2485	 0.2179	 0.2406	 0.2490
	Effector NLP VIGS GFP	10	 0.2224	 0.1876	 0.2136	 0.2229
	Effector NLP VIGS BAK1	11	 0.3111	 0.2872	 0.3049	 0.3115
	Effector NLP VIGS SOBIR1	12	 0.2993	 0.2745	 0.2929	 0.2996
		 	1	2	3	4
 
		 	 	 	 	 
	Effector 91409 VIGS BAK1	5	 *			
	Effector 91409 VIGS SOBIR1	6	 0.2135	 *		
	Effector 96951 VIGS GFP	7	 0.2212	 0.2576	 *	
	Effector 96951 VIGS BAK1	8	 0.1706	 0.2158	 0.2234	 *
	Effector 96951 VIGS SOBIR1	9	 0.2030	 0.2422	 0.2490	 0.2054
	Effector NLP VIGS GFP	10	 0.1699	 0.2153	 0.2229	 0.1728
	Effector NLP VIGS BAK1	11	 0.2757	 0.3061	 0.3115	 0.2776
	Effector NLP VIGS SOBIR1	12	 0.2626	 0.2941	 0.2996	 0.2645
		 	5	6	7	8
 
		 	 	 	 	 
	Effector 96951 VIGS SOBIR1	9	 *			
	Effector NLP VIGS GFP	10	 0.2049	 *		
	Effector NLP VIGS BAK1	11	 0.2988	 0.2764	 *	
	Effector NLP VIGS SOBIR1	12	 0.2865	 0.2633	 0.3394	 *
		 	9	10	11	12
 
 

 
 

Least significant differences of predictions (0.1% level)
 
		 	 	 	 	 
	Effector 90017 VIGS GFP	1	 *			
	Effector 90017 VIGS BAK1	2	 0.3097	 *		
	Effector 90017 VIGS SOBIR1	3	 0.3377	 0.2986	 *	
	Effector 91409 VIGS GFP	4	 0.3482	 0.3104	 0.3384	 *
	Effector 91409 VIGS BAK1	5	 0.2915	 0.2451	 0.2797	 0.2922
	Effector 91409 VIGS SOBIR1	6	 0.3397	 0.3009	 0.3297	 0.3403
	Effector 96951 VIGS GFP	7	 0.3482	 0.3104	 0.3384	 0.3488
	Effector 96951 VIGS BAK1	8	 0.2943	 0.2485	 0.2827	 0.2951
	Effector 96951 VIGS SOBIR1	9	 0.3283	 0.2879	 0.3179	 0.3289
	Effector NLP VIGS GFP	10	 0.2938	 0.2479	 0.2822	 0.2944
	Effector NLP VIGS BAK1	11	 0.4110	 0.3794	 0.4028	 0.4115
	Effector NLP VIGS SOBIR1	12	 0.3954	 0.3626	 0.3869	 0.3958
		 	1	2	3	4
 
		 	 	 	 	 
	Effector 91409 VIGS BAK1	5	 *			
	Effector 91409 VIGS SOBIR1	6	 0.2821	 *		
	Effector 96951 VIGS GFP	7	 0.2922	 0.3403	 *	
	Effector 96951 VIGS BAK1	8	 0.2254	 0.2851	 0.2951	 *
	Effector 96951 VIGS SOBIR1	9	 0.2682	 0.3200	 0.3289	 0.2713
	Effector NLP VIGS GFP	10	 0.2245	 0.2845	 0.2944	 0.2283
	Effector NLP VIGS BAK1	11	 0.3642	 0.4044	 0.4115	 0.3668
	Effector NLP VIGS SOBIR1	12	 0.3469	 0.3885	 0.3958	 0.3494
		 	5	6	7	8
 
		 	 	 	 	 
	Effector 96951 VIGS SOBIR1	9	 *			
	Effector NLP VIGS GFP	10	 0.2707	 *		
	Effector NLP VIGS BAK1	11	 0.3948	 0.3652	 *	
	Effector NLP VIGS SOBIR1	12	 0.3785	 0.3478	 0.4483	 *
		 	9	10	11	12
 
 


Table S3. Regression analysis of VIGS experimental data. Predictions from the regression model and least significant differences of predictions at three significance levels (5%, 1% and 0.1%) are shown.
